# Drivers of HIV-1 drug resistance to non-nucleoside reverse-transcriptase inhibitors (NNRTIs) in nine southern African countries: a modelling study

**DOI:** 10.1186/s12879-021-06757-6

**Published:** 2021-10-07

**Authors:** Julien Riou, Carole Dupont, Silvia Bertagnolio, Ravindra K. Gupta, Roger D. Kouyos, Matthias Egger, Christian L. Althaus

**Affiliations:** 1 Institute of Social and Preventive Medicine (ISPM), University of Bern, Mittelstrasse 43, 3012 Bern, Switzerland; 2 HIV/Hepatitis/STI Department, World Health Organization, Geneva, Switzerland; 3 Department of Infection, University College London, London, UK; 4 Africa Health Research Institute, Durban, South Africa; 5 Division of Infectious Diseases and Hospital Epidemiology, University Hospital Zurich, Zurich, Switzerland; 6 Institute of Medical Virology, University of Zurich, Zurich, Switzerland; 7 Centre for Infectious Disease Epidemiology and Research (CIDER), University of Cape Town, Cap Town, South Africa; 8 Population Health Sciences, Bristol Medical School, University of Bristol, Bristol, UK

**Keywords:** HIV drug resistance, Epidemiology, Southern Africa, Antiretroviral therapy, Health system science, Modelling

## Abstract

**Introduction:**

The rise of HIV-1 drug resistance to non-nucleoside reverse-transcriptase inhibitors (NNRTI) threatens antiretroviral therapy's long-term success (ART). NNRTIs will remain an essential drug for the management of HIV-1 due to safety concerns associated with integrase inhibitors. We fitted a dynamic transmission model to historical data from 2000 to 2018 in nine countries of southern Africa to understand the mechanisms that have shaped the HIV-1 epidemic and the rise of pretreatment NNRTI resistance.

**Methods:**

We included data on HIV-1 prevalence, ART coverage, HIV-related mortality, and survey data on pretreatment NNRTI resistance from nine southern Africa countries from a systematic review, UNAIDS and World Bank. Using a Bayesian hierarchical framework, we developed a dynamic transmission model linking data on the HIV-1 epidemic to survey data on NNRTI drug resistance in each country. We estimated the proportion of resistance attributable to unregulated, off-programme use of ART. We examined each national ART programme's vulnerability to NNRTI resistance by defining a fragility index: the ratio of the rate of NNRTI resistance emergence during first-line ART over the rate of switching to second-line ART. We explored associations between fragility and characteristics of the health system of each country.

**Results:**

The model reliably described the dynamics of the HIV-1 epidemic and NNRTI resistance in each country. Predicted levels of resistance in 2018 ranged between 3.3% (95% credible interval 1.9–7.1) in Mozambique and 25.3% (17.9–33.8) in Eswatini. The proportion of pretreatment NNRTI resistance attributable to unregulated antiretroviral use ranged from 6% (2–14) in Eswatini to 64% (26–85) in Mozambique. The fragility index was low in Botswana (0.01; 0.0–0.11) but high in Namibia (0.48; 0.16–10.17), Eswatini (0.64; 0.23–11.8) and South Africa (1.21; 0.83–9.84). The combination of high fragility of ART programmes and high ART coverage levels was associated with a sharp increase in pretreatment NNRTI resistance.

**Conclusions:**

This comparison of nine countries shows that pretreatment NNRTI resistance can be controlled despite high ART coverage levels. This was the case in Botswana, Mozambique, and Zambia, most likely because of better HIV care delivery, including rapid switching to second-line ART of patients failing first-line ART.

**Supplementary Information:**

The online version contains supplementary material available at 10.1186/s12879-021-06757-6.

## Introduction

The rising prevalence of HIV-1 drug resistance is threatening the success of combination antiretroviral therapy (ART) programmes in southern Africa and beyond. This region has the highest burden of HIV-1 infection, accounting for about 30% of persons living with HIV-1 (PLHIV) globally [1, 2]. Since the early 2000s, first-line ART consisting of one non-nucleoside reverse-transcriptase inhibitor (NNRTI) and two nucleoside reverse-transcriptase inhibitors (NRTIs) was introduced through national access programmes [3]. As of 2019, an estimated 72% of adults living with HIV-1 in the region were treated with ART [4].

A substantial increase in HIV-1 drug resistance followed the scale-up of ART [5], compounded by limited access to resistance testing and low rates of switching to second-line ART (based on protease inhibitors) [6]. Drug resistance is associated with poor virological, immunological, and clinical outcomes [7–9]. Defined as the proportion of PLHIV with resistance mutations before or at the time of ART initiation, the prevalence of pretreatment resistance is commonly used to monitor resistance in a population. The prevalence of pretreatment NNRTI resistance in 2016 was estimated at 11.0% in southern Africa [10]. While NNRTIs are being replaced by the integrase inhibitor dolutegravir in this region and elsewhere, NNRTIs will likely remain an essential drug for the management of HIV-1, considering the adverse effects and unconfirmed safety concerns associated with DTG [11–13]. Also, there is a risk of DTG resistance, such as in PLHIV who switch to DTG-based ART with an unsuppressed viral load [14, 15]. A better understanding of the factors that led to the level of NNRTI resistance observed in a country could be critical to control future DTG resistance.

Several factors influence the growth of pretreatment NNRTI resistance. First, de novo resistance mutations can emerge in PLHIV on first-line ART who do not suppress viral replication [16, 17]. Of note, multi-class drug resistance is common even with the modern nucleoside reverse transcriptase inhibitor (NRTI) tenofovir [18, 19]. Resistance to NNRTIs is facilitated by their long half-lives and the fact that single nucleotide substitutions can result in high-level NNRTI resistance and NNRTI cross-resistance [16, 20, 21]. Virological failure may be precipitated by suboptimal HIV service delivery, including poor adherence support and retention on ART or unreliable drug supply, by inappropriate, off-programme antiretroviral use or single-drug regimens used for the prevention of mother-to-child transmission (PMTCT) [22]. Second, the many PLHIV on first-line ART induce a selective advantage of drug-resistant HIV-1 strains over sensitive strains, with increased transmission during virological failure [5, 17, 23]. Third, the dynamic of the HIV-1 epidemic in a country might influence exposure to NNRTI-resistant strains, and the evolution of pretreatment NNRTI resistance. Mathematical models can contribute to a better understanding of the mechanisms that shape the HIV-1 epidemic and the rise of pretreatment NNRTI resistance in a country and thus inform policy. We fitted a dynamic transmission model of HIV-1 to data on the HIV-1 epidemic and pretreatment NNRTI resistance from nine countries in southern Africa using a hierarchical Bayesian framework. For each country, we estimated the evolution of pretreatment NNRTI resistance with time. We quantified the proportion of pretreatment resistance that can be attributed to off-programme antiretroviral use. We estimated an index of fragility regarding NNRTI resistance for each national ART programme, thus quantifying a country programme's ability to minimise pretreatment NNRTI resistance. We then explored associations between the fragility index and characteristics of health systems.

## Methods

### Data

We updated a systematic review of pretreatment drug resistance surveys in HIV-1-infected adults (aged > 15 years) in low- and middle-income countries [10]. The review included publications and abstracts published from Jan 1, 2001, to Dec 31, 2016, and unpublished data from surveys supported by the World Health Organization (WHO). We updated the review for the period from Jan 1, 2017, to Jul 31, 2019, in PubMed, Embase and WHO reports using the same search strategy (Additional file 1: Appendix pp 2–4).

We screened studies for eligibility based on title and abstract. Eligible studies had to report original survey data about the proportion of NNRTI resistance in a sample of adults (> 15 years) before or at the time of ART initiation. The data had to be collected between 2000 and 2018 in one or several of the nine countries of southern Africa (Botswana, Eswatini, Lesotho, Malawi, Mozambique, Namibia, South Africa, Zambia, and Zimbabwe). Two independent reviewers (JR and CD) assessed the full manuscripts of potentially eligible studies. We used data from 56 surveys of pretreatment NNRTI resistance from southern Africa that were identified in the original review. The updated search yielded 1,039 items, leading to eight additional eligible studies. In total, we thus included data from 64 surveys of pretreatment NNRTI resistance in nine countries (56 from the original review and 8 from the update), for a total of 14,567 individuals.

For each country, we obtained data describing the HIV-1 epidemic for each year from 2000 to 2018: (i) the number of adults living with HIV, (ii) the number of adults living with HIV-1 receiving ART, (iii) the number of AIDS-related deaths among adults, and (iv) the size of the adult population. Data used in this analysis were not raw data from individual patients but aggregated data [4, 24]. They were aggregated at the country level as reported by UNAIDS and the World Bank.

### Model

**Fig. 1:**
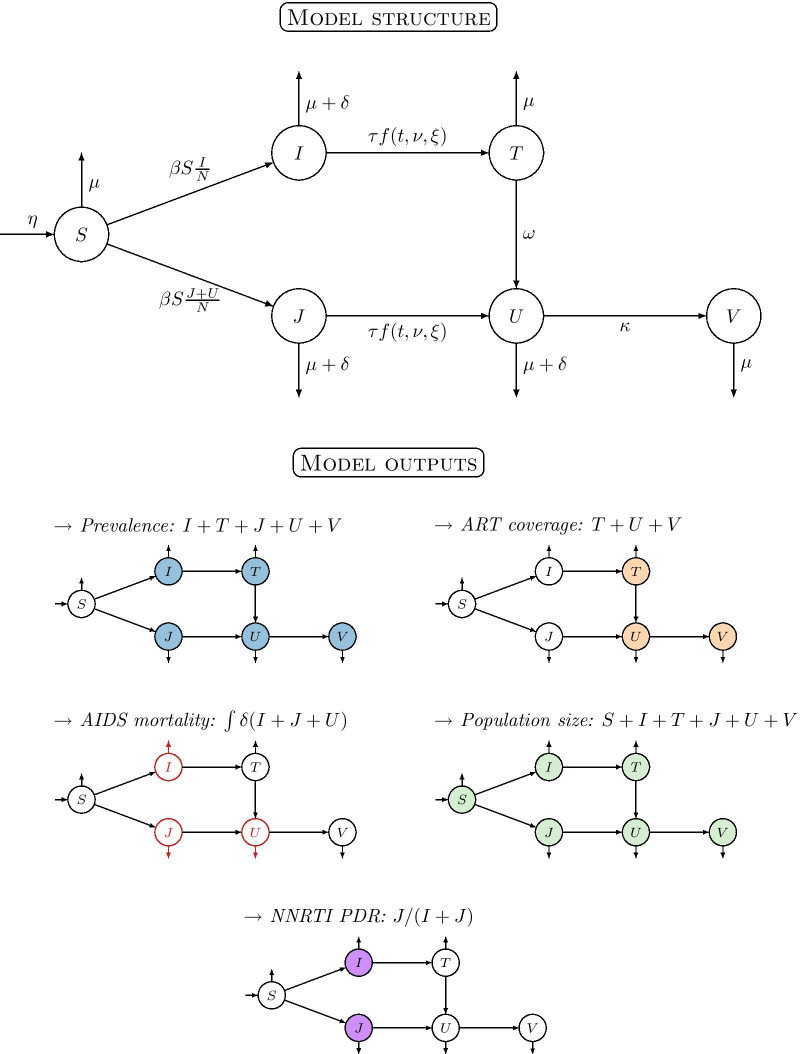
Structure of the dynamic model of HIV-1 transmission, ART rollout and resistance emergence (upper panel), and model outputs with colours corresponding to Figs. 2 and 3 (lower panel). The population is split into six compartments: susceptible to HIV-1 ($S$); infected with a drug-sensitive strain and either untreated ($I$) or treated with first-line ART ($T$); infected with a drug-resistant strain and either untreated ($J$), treated with first-line ART ($U$) or treated with second-line ART ($V$). The model has ten parameters: transmission rate $\beta$; maximal treatment rate $\tau$ scaled by a sigmoid function $f\left(t,\nu ,\xi \right)$ (controlled by two parameters for shift $\nu$ and slope $\xi$); rate at which de novo NNRTI resistance emerges during treatment ($\omega$); rate of switching to second-line ART ($\kappa$); rate of AIDS-related mortality ($\delta$); rate of background mortality ($\mu$); rate of population growth ($\eta$); and the initial proportion of NNRTI resistance $\iota$ (not shown)

We developed a dynamic transmission model linking the country-specific data on the HIV-1 epidemic to survey data on NNRTI drug resistance in each country. The model consists of a system of ordinary differential equations with six compartments (Fig. 1), with the following features:

- **HIV-1 transmission**. The force of infection of drug-sensitive and drug-resistant HIV-1 strains depends on the transmission rate $\beta$. We assumed that only individuals without treatment (compartments $I$ and $J$) or who fail first-line treatment (compartment $U$) can transmit HIV-1 [25], and that there is no fitness cost associated with the transmission of drug-resistant strains [26].
- **First-line NNRTI-based ART**. Untreated individuals initiate first-line ART at a time-dependent rate $\tau f\left(t,\nu ,\xi \right)$, where the maximal treatment rate $\tau$ is scaled by a sigmoid function $f\left(t,\nu ,\xi \right)$ to reflect the progressive rollout of ART.
- **Emergence of de novo NNRTI resistance**. HIV-1 infected adults on first-line ART can acquire de novo NNRTI resistance at rate $\omega$.
- **Initial levels of pretreatment NNRTI resistance.** We defined the starting date of the model in 1999, before the implementation of ART programmes. We describe the level of pretreatment NNRTI resistance at that date by an intercept parameter $\iota$. Similar to the intercept in a general linear model, this parameter describes the proportion of resistance not explained by the rollout of ART within ART programmes, fixing the treatment rate at 0.
- **Second-line NNRTI-free ART**. Individuals who fail first-line ART due to NNRTI resistance may be switched to second-line NNRTI-free ART regimens at rate $\kappa$.
- **Demography**. New individuals enter the adult population (> 15 years) at rate $\eta$. All individuals can die from background mortality at rate $\mu$, which we fix to the inverse of the life expectancy in adults in each country. HIV-1 infected individuals without treatment (compartments $I$ and $J$) or who fail first-line ART (compartment $U$) die from AIDS-related mortality at rate $\delta$.

We used the model to estimate two country-level indicators of pretreatment NNRTI resistance:

- **Pretreatment NNRTI resistance unrelated to ART programmes.** This indicator is defined as $\iota /PD{R}_{2018}$, where the numerator is the intercept of pretreatment NNRTI resistance in the country, and the denominator the predicted level of pretreatment NNRTI resistance in 2018. It quantifies the relative impact of unregulated, off-programme antiretroviral use or single-drug regimens used for the prevention of mother-to-child transmission (PMTCT) on pretreatment NNRTI resistance in a country.
- **Fragility of national ART programmes.** This indicator reflects the ability of a country's ART programme to minimise the rise in pretreatment drug resistance. It is measured by the ratio of $\omega$, i.e. the rate of occurrence of NNRTI resistance in adults on first-line ART over $\kappa$, i.e. the rate of switching to second-line ART. It is a proxy measure for the growth of pretreatment NNRTI resistance in a country resulting from first-line ART rollout. It is not dependent on the scale and timing of the ART rollout and directly comparable across countries. High values (relatively slow switch to second-line ART and relatively rapid occurrence of NNRTI resistance) indicate fragile ART programmes. Low values (relatively rapid switch to second-line ART and relatively slow occurrence of NNRTI resistance) describe ART programmes that are resilient against the emergence of pretreatment NNRTI resistance. In this framework, differences in delays to virological failure detection across countries would translate into differences in the fragility index that we propose.

### Bayesian hierarchical framework

We implemented the model in the Bayesian statistical software Stan 2.18.2 [27]. The ODEs for each country were numerically integrated in parallel, with a starting date in 1999. There were nine free model parameters $\{\beta ,\tau ,\xi ,\nu ,\omega ,\kappa ,\delta ,\eta ,\iota \}$ and one fixed parameter $\mu$ for each country. We imposed a hierarchical structure on the parameters related to NNRTI resistance ($\omega$ and $\iota$). The other parameters were independently estimated for each country. We selected weakly-informative prior distributions for all free parameters and conducted prior predictive checks to ensure that the chosen priors limited the range of explored parameter space to sensible values [28]. By fitting the full hierarchical model to all available data, we obtained the joint posterior distribution for the nine free model parameters in each country. Further details are available in Additional file 1: Appendix, pp 4–8.

### Socio-economic variables

We explored associations between the fragility index of ART programmes to pretreatment NNRTI resistance and seven country-level variables measuring characteristics of health systems and countries: (i) pregnant women who received NNRTIs for PMTCT as a proportion of HIV-1 adult prevalence, (ii) total health expenditure per capita (in $ purchasing power parity), (iii) the proportion of international donor funding in total health expenditure, (iv) proportion of out-of-pocket health expenditure, (v) gross national income per capita (in $), (vi) proportion of rural population, (vii) proportion of unemployed. Data were retrieved from the World Bank or UNAIDS [4, 24]. We used the mean over the years 2000 to 2018 for this analysis. We computed Spearman's rank correlation coefficients between the estimated indicator of the response of ART programmes to pretreatment NNRTI resistance and each of the seven country-level covariates.

### Funding

National Institute of Allergy and Infectious Diseases (grant 5U01-AI069924-05) and Swiss National Science Foundation (grant 174281). Study funders had no role in study design, data analysis, interpretation of results, or writing of the report.

## Results

**Fig. 2:**
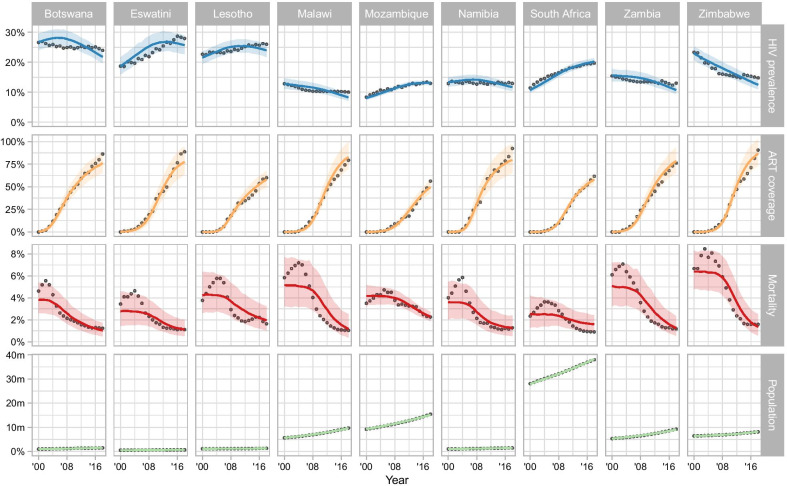
Model fit for HIV-1 prevalence, ART coverage, mortality, and population size for nine countries of southern Africa, 2000 to 2018. Circles represent data obtained from UNAIDS and the World Bank. Lines and shaded areas correspond to model predictions (median posterior and 95% credible interval)

**Fig. 3:**
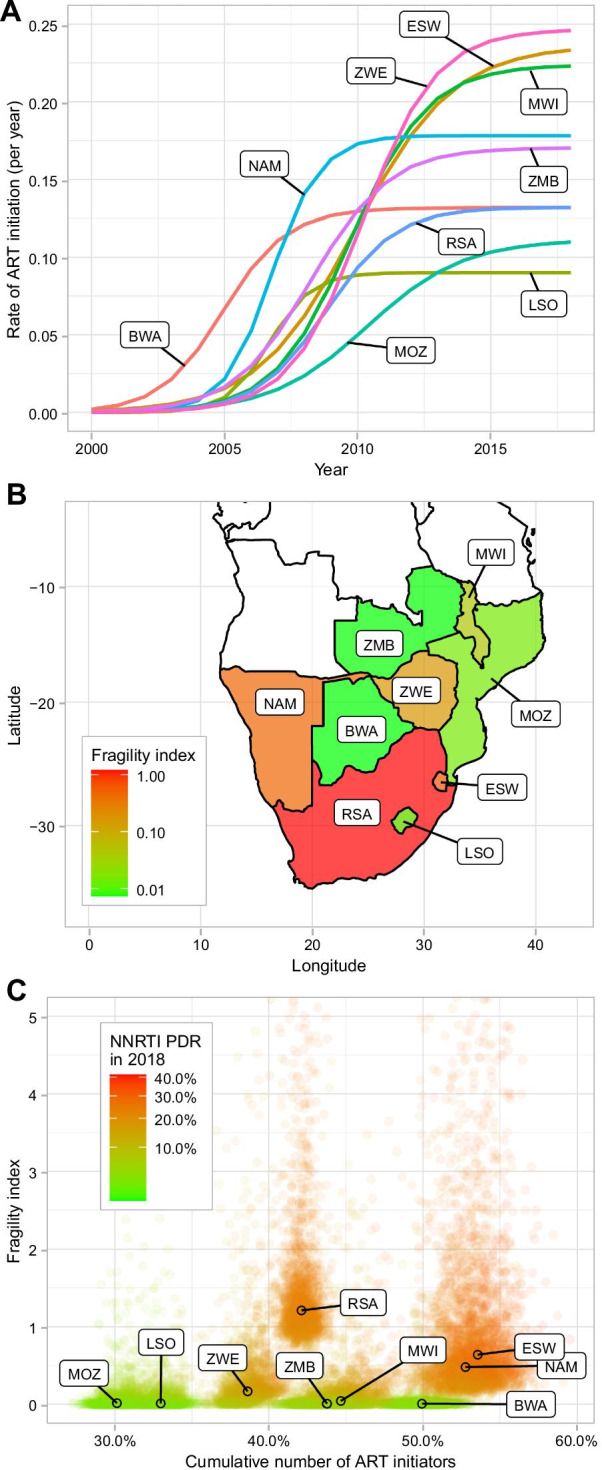
**A** Visualising ART rollout: change in the rate of ART initiation (per year) between 2000 and 2018 as estimated from the model. **B** Estimates of the fragility index of ART programmes by country. **C** Three-way scatter plot between the posterior samples of: the fragility index of ART programmes (y-axis), the proportion of all persons living with HIV that initiated ART over the period 2000–2018 (x-axis) and the predicted levels of pretreatment NNRTI resistance (PDR) in 2018 (colour gradient). *BWA* Botswana, *ESW* Eswatini, *LSO* Lesotho, *MWI* Malawi, *MOZ* Mozambique, *NAM* Namibia, *RSA* Republic of South Africa, *ZMB* Zambia, *ZWE* Zimbabwe

The model reflected the general trends of HIV-1 prevalence, ART coverage, AIDS-related mortality and population size in each country for the period 2000–2018 (Fig. 2). The levels and trends of HIV-1 adult prevalence varied by country. Higher prevalences were observed in Botswana, Eswatini, and Lesotho and lower, decreasing prevalences in Malawi, Zambia, and Zimbabwe. The model fitted the course of AIDS-related mortality less well, but it captured the magnitude and overall trends of mortality in each country. The timing of the ART rollout also varied across countries (Figs. 2, 3A). It occurred first in Botswana and Namibia, quickly followed by most of the other countries, except Mozambique, which rolled out ART later and at a lower rate. The peak rate at which patients initiated first-line ART was highest in Zimbabwe and Eswatini, and lowest in Mozambique and Lesotho (Fig. 3A).

**Fig. 4:**
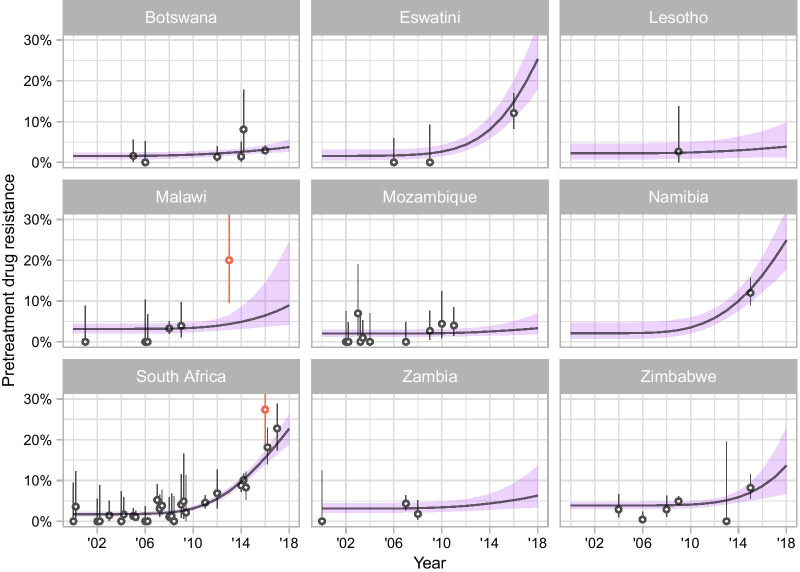
Model fit for the prevalence of pretreatment NNRTI resistance in nine countries of southern Africa from 2000 to 2018. Circles represent survey results with 95% credible intervals. Lines and shaded areas correspond to model predictions (median posterior and 95% credible interval)

Linking the dynamics of HIV-1 transmission, treatment and mortality to survey data on pretreatment NNRTI resistance, the model estimated the rise of NNRTI resistance in every country (Fig. 4). There was good agreement between the estimated trajectory of pretreatment NNRTI resistance between 2000 and 2018 and the survey data. There were only two outliers (defined as survey estimates with confidence intervals that did not overlap with modelled estimates). One outlying study was from Malawi among persons with acute HIV-1 infection [29] and the other one from South Africa among sex workers [30]. Model-predicted levels of pretreatment NNRTI resistance in 2018 ranged between 3.3% (95% credible interval [CrI] 1.9 to 7.1%) in Mozambique and 25.3% (17.9 to 33.8%) in Eswatini (Table 1). Large uncertainty intervals reflected the scarcity of recent survey data in Eswatini, Lesotho, Malawi, Namibia, and Zambia.

**Table 1:** Country-level estimates of the main aspects of ART rollout and pretreatment NNRTI resistance in southern Africa

Country	Timing of ART rollout, year (95% CrI)	Intensity of ART rollout, per year (95% CrI)	NNRTI PDR in 2018 (%) (95% CrI)	NNRTI PDR in 2018 unrelated to ART programmes (%) (95% CrI)	Proportion of NNRTI PDR in 2018 unrelated to ART programmes (%) (95% CrI)	Fragility index regarding NNRTI PDR of ART programmes (95% CrI)
Botswana	2005 (2004–2006)	0.13 (0.12–0.15)	3.7% (2.6–5.6)	1.5% (0.7–2.4)	42% (15–2)	0.01 (0.00–0.11)
Eswatini	2009 (2009–2009)	0.24 (0.18–0.29)	25.3% (17.9–33.7)	1.6% (0.5–3.0)	6% (2–14)	0.64 (0.23–11.8)
Lesotho	2007 (2006–2007)	0.09 (0.082–0.099)	3.9% (1.3–9.8)	2.2% (0.8–4.6)	62% (20–86)	0.01 (0.00–0.70)
Malawi	2009 (2009–2009)	0.22 (0.18–0.28)	8.9% (4.2–24.4)	3.2% (2.1–4.4)	37% (11–72)	0.04 (0.00–1.30)
Mozambique	2009 (2009–2009)	0.11 (0.094–0.13)	3.3% (1.9–6.9)	2.0% (1.2–3.0)	65% (26–84)	0.01 (0.00–0.70)
Namibia	2007 (2006–2008)	0.18 (0.15–0.21)	24.9% (17.8–32.2)	2.1% (0.8–4.7)	8% (3–22)	0.48 (0.15–11.2)
South Africa	2009 (2009–2009)	0.13 (0.12–0.14)	22.7% (18.9–26.1)	1.7% (1.3–2.3)	8% (5–10)	1.21 (0.83–9.84)
Zambia	2008 (2007–2009)	0.17 (0.14–0.21)	6.3% (3.5–13.5)	3.1% (2.1–4.4)	50% (21–80)	0.01 (0.00–0.27)
Zimbabwe	2009 (2009–2009)	0.25 (0.2–0.32)	13.7% (6.8–22.9)	3.9% (3.0–4.7)	28% (15–60)	0.17 (0.00–2.65)

*PDR* pretreatment drug resistance

The timing of ART rollout refers to the date at which 50% of the maximum treatment rate is reached. The intensity of ART rollout refers to the peak treatment rate per year. NNRTI PDR in 2018 is the model-predicted prevalence of NNRTI PDR. Posterior medians with 95% credibility intervals are shown

The absolute prevalence of pretreatment NNRTI resistance in 2018 that was estimated not to be attributable to the functioning of the ART programmes ranged from 1.5% in Botswana (95%CrI 0.7–2.4) and 1.5% in Eswatini (95%CrI 0.5–3.0) to 3.9% (95%CrI 3.1–4.8) in Zimbabwe (Table 1). When expressing this prevalence in relative terms as a proportion of the overall prevalence of pretreatment NNRTI resistance, the proportion was small in Eswatini, Namibia, and the Republic of South Africa, ranging from 6% (95%CrI 2–14) in Eswatini to 8% (95%CrI 3–22) in Namibia. In contrast, overall pretreatment NNRTI resistance appears to have been strongly influenced by unregulated off-programme antiretroviral use in Zambia, Lesotho, and Mozambique, ranging from 50% (95%CrI 22–80) in Zambia to 64% (95%CrI 26–85) in Mozambique (Table 1).

Country-specific estimates of ART programmes' fragility regarding pretreatment NNRTI resistance showed marked differences across countries (Table 1, Fig. 3B). In Botswana, Lesotho, Mozambique, and Zambia, fragility was low, with values close to 0, indicating that a rapid switch to second-line ART compensated the number of patients acquiring de novo NNRTI resistance during first-line ART. Switching to second-line ART may thus have prevented the further spread of NNRTI resistance in these countries. Conversely, the national ART programmes in Eswatini, Namibia, and the Republic of South Africa were more fragile, with index values ranging from 0.48 (95% CrI 0.16–10.17) in Namibia to 1.19 (95%CrI 0.834–6.98) in the Republic of South Africa. The combination of a high fragility of ART programmes regarding pretreatment NNRTI resistance and high levels of ART coverage was associated with a sharp increase in pretreatment NNRTI resistance following the scale-up of ART in these three countries (Fig. 3C).

The variation between countries regarding the response to pretreatment NNRTI resistance was partly explained by differences in country characteristics (Table 2). For example, lower fragility regarding pretreatment NNRTI resistance was found in countries with higher levels of international donor funding (as a proportion of total health expenditure). Fragility was higher in countries with higher levels of total health expenditure. Of note, we did not find evidence of an association between fragility regarding NNRTI resistance and the intensity of PMTCT programmes.

**Table 2:** Country-level characteristics associated with the fragility of ART programmes in southern Africa regarding pretreatment drug resistance to non-nucleoside reverse transcriptase inhibitors (NNRTI)

Country-level covariate	Correlation^a^
**Gross national income per capita** (US$)	0.28 (− 0.08 to 0.70)
**Current health expenditure per capita** (US$)	0.53 (0.17 to 0.85)
**External donor funding** (proportion of current health expenditure)	− 0.45 (− 0.80 to − 0.10)
**Out-of-pocket expenditure** (proportion of current health expenditure)	0.02 (− 0.43 to 0.40)
**Rural population** (proportion of total population)	− 0.17 (− 0.55 to 0.18)
**PMTCT** (proportion of total prevalence^b^)	0.28 (− 0.15 to 0.63)
**Unemployment** (proportion of total labour force)	0.22 (− 0.18 to 0.67)

PMTCT, prevention of mother to child transmission

^a^Spearman's rank correlation coefficient between the posterior samples of the indicator of vulnerability to pretreatment NNRTI resistance within ART access programmes and each covariate (median and 95% credible interval)

^b^The proportion of pregnant women who received NNRTIs for PMTCT as a proportion of HIV-1 adult prevalence

## Discussion

This study provides a detailed analysis of the rise of pretreatment NNRTI resistance in nine countries of southern Africa for the period 2000 to 2018. We used a dynamic transmission model to link the local characteristics of HIV-1 transmission, ART scale-up and mortality at the country level to data from a systematic review of pretreatment NNRTI resistance in adults. By including all the relevant information from these countries, we could examine the dynamics and drivers of pretreatment NNRTI resistance in a region heavily affected by the HIV-1 pandemic. In particular, we used the model to estimate two country-level indicators of pretreatment NNRTI resistance: the proportion of resistance related to the unregulated, off-programme use of antiretrovirals, and the fragility of national ART programmes regarding the threat of resistance.

The increase in pretreatment NNRTI resistance between 2000 and 2018 differed across the nine countries. It was driven by country-level differences in both the ART rollout's timing and intensity and ART programmes' fragility. While the evolutionary pressure exerted by NNRTI-based first-line ART obtained through ART programmes is a necessary condition for the selection of NNRTI resistance mutations [16], other intricate factors influence the level of pretreatment NNRTI resistance in a country. The comparison between Botswana and Eswatini illustrates this situation. The early scale-up of ART in Botswana and the later but more intense rollout in Eswatini led to a similar number of ART initiations relative to the two countries' populations. Also, the model estimated pretreatment NNRTI resistance levels attributable to unregulated, off-programme antiretroviral at around 1.5% for both countries. Yet, the trajectories of NNRTI resistance differed widely, growing to an estimated 25.3% in Eswatini in 2018 compared to 3.8% in Botswana. This difference probably resulted from a loss of control of the ART programme in Eswatini. Indeed, the indicator for the national ART programme's fragility was 0.65 in Eswatini compared to 0.01 in Botswana. Of note, the response estimated for the South African ART programme was also poor. In South Africa, pretreatment NNRTI resistance levels were high (estimated at 22.5%) despite the ART rollout's average intensity.

Our study indicates that the countries of southern Africa cluster into three groups. In a first group, comprised of Botswana, Lesotho, Mozambique, and Zambia, pretreatment NNRTI resistance remained below 10% in 2018, associated with a low fragility of ART programmes. The good control exerted within ART programmes prevented the rise of pretreatment NNRTI resistance at the population level, even where ART was scaled-up early and massively, such as Botswana (Fig. 3A). Residual resistance was primarily attributable to unregulated off-programme antiretroviral use. In contrast, pretreatment resistance rose dramatically in the second group, comprising Eswatini, Namibia, and South Africa. Off-programme antiretroviral use was relatively less important in these countries, with the increase mostly driven by the combination of a massive scale-up of ART and fragile ART programmes. The third group of countries included Malawi and Zimbabwe, with pretreatment resistance levels in 2018 around 10%, related to both an intermediate level of fragility of ART programmes and some contribution by off-programme antiretroviral use.

The indicator for the fragility of ART programmes can be interpreted as a composite indicator of the level of control achieved in a country during ART rollout, capturing the quality of ART delivery [22], including adherence support and virologic monitoring [16, 20, 30, 31]. The modification of ART eligibility towards earlier treatment initiation, may also have contributed to drug resistance emergence [15]. Earlier treatment is associated with higher levels of non-adherence, and the scale-up of treatment programmes may have strained fragile health systems [32]. International donor funding was associated with a lower fragility of ART programmes. This may be explained by the additional, targeted funding of ART programmes, coupled with the programmatic support and monitoring by international donors. Conversely, higher levels of total health expenditure was associated with a worse response of ART programmes to the threat of pretreatment NNRTI resistance. The rapid scale-up of ART in some countries may have overburdened the health system and affected patient management. Although PMTCT has been proposed as an essential driver of NNRTI resistance [22], we found little evidence for an association between PMTCT coverage and fragility. This may be due to the relatively homogeneous uptake of PMTCT across countries of the region.

Our study's main strength is the application of a dynamic transmission model, which describes how the emergence of NNRTI drug resistance, together with the dynamics of HIV-1 transmission, ART scale-up and mortality produced disparate trends in NNRTI resistance across southern Africa. Fitted to country-level data using Bayesian inference, the model allowed for the full propagation of uncertainty and relied on a few assumptions. We focused on mechanisms that could be informed by data available for all countries during the period of interest. While the model captured the main trends of the HIV-1 epidemic in each country, its relatively simple structure ignored some dimensions such as age, gender, acute infections and disease progression. However, adding more dimensions would have increased the complexity of a model that is already computationally expensive to fit due to its hierarchical nature. These considerations meant that we did not consider data on previous exposure to PMTCT, off-prescription use of ART or undocumented treatment discontinuation. Rather, these mechanisms were estimated together by considering the proportion of pretreatment NNRTI resistance that is unrelated to the ART rollout.

Another limitation concerns the analysis of country-level data, exposing the results to ecological bias, and ignoring potential within-country heterogeneity. This choice was dictated by the scale of available data and the fact that HIV-related health policies are implemented at the national level. More data on NNRTI resistance, treatment adherence, previous exposure to ART, loss to follow-up, virological failure, and ART coverage for first and second-line treatment, collected systematically at the country level or at a lower scale, would be necessary to improve the precision of the estimates. A last limitation concerns the analysis of the characteristics associated with the fragility of national ART programmes. This analysis was exploratory and based on a small number of countries and indicators.

In conclusion, the rollout of ART in southern Africa was followed by increasing levels of pretreatment NNRTI resistance. This increase has been heterogeneous across countries of the region. The between-country comparison shows that resistance can be controlled despite high levels of ART coverage. Our results suggest that the fragility of ART programmes regarding pretreatment NNRTI resistance is associated with features of the healthcare system at the national level. Further research is needed to determine more precisely what organisational features allowed the control of pretreatment NNRTI resistance in countries like Botswana, Lesotho, Mozambique, and Zambia. Our results suggest that resistance, once emerged, can be controlled even if the drug is made used very widely. They have implications for the ongoing rollout of dolutegravir in southern Africa. Concerns about drug resistance should not lead to restrictions of the use of dolutegravir, which has a high genetic barrier to resistance [33] or the use of NNRTIs where it continues to be appropriate, but rather to interventions aimed at controlling the emergence and spread of resistance.

## Supplementary Information

**Additional file 1.** Additional details about the data and the model.
