# Correction to: Drivers of HIV-1 drug resistance to non-nucleoside reverse-transcriptase inhibitors (NNRTIs) in nine southern African countries: a modelling study

**DOI:** 10.1186/s12879-021-06791-4

**Published:** 2021-10-25

**Authors:** Julien Riou, Carole Dupont, Silvia Bertagnolio, Ravindra K. Gupta, Roger D. Kouyos, Matthias Egger, Christian L. Althaus

**Affiliations:** 1 Institute of Social and Preventive Medicine (ISPM), University of Bern, Mittelstrasse 43, 3012 Bern, Switzerland; 2 HIV/Hepatitis/STI Department, World Health Organization, Geneva, Switzerland; 3 Department of Infection, University College London, London, UK; 4 Africa Health Research Institute, Durban, South Africa; 5 Division of Infectious Diseases and Hospital Epidemiology, University Hospital Zurich, Zurich, Switzerland; 6 Institute of Medical Virology, University of Zurich, Zurich, Switzerland; 7 Centre for Infectious Disease Epidemiology and Research (CIDER), University of Cape Town, Cap Town, South Africa; 8 Bristol Medical School, Population Health Sciences, University of Bristol, Bristol, UK

## Correction to: BMC Infect Dis (2021) 21:1042 https://doi.org/10.1186/s12879-021-06757-6

Following publication of the original article [1], it was noted that the Supplementary file was missing.

Additional file 1 can be found in this Correction article (Additional file 1).

The original article has been corrected as well.

## Supplementary Information

**Additional file 1.** Additional details about the data and the model.

## References

[1] Riou J, Dupont C, Bertagnolio S, Gupta RK, Kouyos RD, Egger M, Althaus CL (2021). Drivers of HIV-1 drug resistance to non-nucleoside reverse-transcriptase inhibitors (NNRTIs) in nine southern African countries: a modelling study. BMC Infect Dis.

