# Supplementary material for: Drivers of HIV-1 drug resistance to non-nucleoside reverse-transcriptase inhibitors (NNRTIs) in nine southern African countries: a modelling study
Source: BMC Infect Dis. 2021 Oct 7;21:1042. doi: 10.1186/s12879-021-06757-6 (PMC8499543; doi:10.1186/s12879-021-06757-6)
Supplement: Supplementary file 1 — Additional file 1. Additional details about the data and the model. [file 12879_2021_6757_MOESM1_ESM.pdf]

Supplementary appendix to:

Dynamics and drivers of HIV-1 drug resistance to non-nucleoside reverse-transcriptase inhibitors in southern Africa

Julien Riou, Carole Dupont, Silvia Bertagnolio, Ravindra K. Gupta, Leigh F. Johnson, Roger D. Kouyos, Matthias Egger, Christian L. Althaus

Contents

1 Data **2**
1.1 Systematic review of NNRTI PDR . . . 2
1.1.1 Initial systematic review . . . 2
1.1.2 Update to the systematic review . . . 2
1.1.3 Included papers . . . 3
1.2 Data on four key indicators . . . 3

2 Additional model description **4**
2.1 HIV transmission dynamics . . . 4
2.2 Hierarchical structure . . . 6
2.3 Statistical inference . . . 6
2.4 Prior predictive checks . . . 7
2.5 Implementation and code . . . 8

1 Data

1.1 Systematic review of NNRTI PDR

1.1.1 Initial systematic review

Survey data on PDR in adults was obtained from a systematic review conducted by [1]. The authors searched for studies in PubMed, Embase and for conference abstracts published between Jan 1, 2001 and Dec 31, 2016, and complemented the systematic review with additional unpublished datasets from WHO-supported surveys of drug resistance. From the items included in the original systematic review, we selected 56 surveys of NNRTI PDR conducted in one of our 9 countries of interest: Botswana, Lesotho, Malawi, Mozambique, Namibia, South Africa, Eswatini, Zambia and Zimbabwe.

1.1.2 Update to the systematic review

Search strategy. The original search strategy not available in the online appendix of the journal article [1]. Therefore, we reproduced the search according to the Methods section (Methods > Search strategy and selection criteria) of the published article, without any language restrictions. We reproduced the search in "All fields" allowing a mapping to thesaurus terms (Mesh and Emtree). We limited time range to the interval January 1, 2017 to July 31, 2019 for PubMed and to August 9, 2019 for Embase. We concentrated on 9 countries of interest.

Table 1: Pubmed search strategy use for the update to the systematic review.

Search	Query	Items found
#1	Search (antiretroviral therapy) AND transmitted drug resistance	686
#2	Search (antiretroviral therapy) AND pretreatment drug resistance	202
#3	Search (antiretroviral therapy) AND (stavudine OR zidovudine OR nevirapine OR efavirenz)	14579
#4	Search (HIV) AND transmitted drug resistance	1204
#5	Search (HIV) AND pretreatment drug resistance	182
#6	Search (HIV) AND antenatal	2924
#7	Search (HIV) AND VCT	775
#8	Search ((genotyp*) AND HIV) AND naive	1720
#9	Search ((genotyp*) AND HIV) AND resistance	5041
#10	Search (((genotyp*) AND HIV) AND resistance) AND primary	673
#11	Search (#1 OR #2 OR #3 OR #4 OR #5 OR #6 OR #7 OR #8 OR #9 OR #10)	23450
#12	Search (Botswana OR Botswan*)	3292
#13	Search (Lesotho OR Lesoth*)	765
#14	Search (Malawi OR Malawi*)	8946
#15	Search (Mozambique OR Mozambi*)	4320
#16	Search (Namibia OR Namibia*)	2110
#17	Search (South-Africa OR South-Africa*)	127230
#18	Search (Eswatini OR Eswatin* OR Swaziland OR Swazi*)	1049
#19	Search (Zambia OR Zambia*)	7343
#20	Search (Zimbabwe OR Zimbabw*)	9138
#21	Search (#12 OR #13 OR #14 OR #15 OR #16 OR #17 OR #18 OR #19 OR #20)	154277
#22	Search (#11 AND #21)	2635
#23	Search (#11 AND #21)	525

Filters: Publication date from 2017/01/01 to 2019/07/31

Identified research items. The search resulted in the identification of 525 items in Pubmed and 913 items in Embase (tables 1 and 2). After deduplication this led to 1,039 items to screen.

Table 2: Embase search strategy use for the update to the systematic review.

Search	Query	Items found
#1	('antiretroviral therapy'/exp OR 'antiretroviral therapy') AND ('transmitted drug resistance'/exp OR 'transmitted drug resistance')	325
#2	('antiretroviral therapy'/exp OR 'antiretroviral therapy') AND 'pretreatment drug resistance'	27
#3	('antiretroviral therapy'/exp OR 'antiretroviral therapy') AND ('stavudine'/exp OR stavudine OR 'zidovudine'/exp OR zidovudine OR 'nevirapine'/exp OR nevirapine OR 'efavirenz'/exp OR efavirenz)	19527
#4	('hiv'/exp OR 'hiv') AND ('transmitted drug resistance'/exp OR 'transmitted drug resistance')	688
#5	('hiv'/exp OR 'hiv') AND 'pretreatment drug resistance'	43
#6	('hiv'/exp OR 'hiv') AND 'antenatal'	3803
#7	('hiv'/exp OR 'hiv') AND ('vct'/exp OR 'vct')	986
#8	'genotyp*' AND ('hiv'/exp OR 'hiv') AND 'naive'	2781
#9	'genotyp*' AND ('hiv'/exp OR 'hiv') AND ('resistance'/exp OR 'resistance')	7223
#10	'genotyp*' AND ('hiv'/exp OR 'hiv') AND ('resistance'/exp OR 'resistance') AND 'primary'	1000
#11	#1 OR #2 OR #3 OR #4 OR #5 OR #6 OR #7 OR #8 OR #9 OR #10	31057
#12	'botswana'/exp OR botswana OR botswan*	4321
#13	'lesotho'/exp OR lesotho OR lesoth*	1002
#14	'malawi'/exp OR malawi OR malawi*	11113
#15	'mozambique'/exp OR mozambique OR mozambi*	5633
#16	'namibia'/exp OR namibia OR namibia*	2688
#17	'south africa'/exp OR 'south africa' OR 'south africa*'	194504
#18	eswatini OR eswatin* OR 'swaziland'/exp OR swaziland OR swazi*	1846
#19	'zambia'/exp OR zambia OR zambia*	9661
#20	'zimbabwe'/exp OR zimbabwe OR zimbabw*	13144
#21	#12 OR #13 OR #14 OR #15 OR #16 OR #17 OR #18 OR #19 OR #20	230881
#22	#11 AND #21	4199
#23	#22 AND (2017:py OR 2018:py OR 2019:py)	913

Screening and inclusion. These 1,039 items were independently screened with title and abstract by two reviewers, CD and JR. The objective was to identify relevant studies according to our inclusion criteria: studies reporting original results on NNRTI PDR in adults in one of the included countries. This step led to the identification of 32 items that were then read by the two reviewers to confirm the inclusion criteria. This second step led to the inclusion of 8 additional studies.

Data extraction. Extracted variables included, for each study i, the number of ART initiators with NNRTI resistance mutations $\mathbb{E}_i$, the sample size $\mathbb{N}_i$ and the median year of sampling $\mathbb{T}_i$.

1.1.3 Included papers

In total, we included 64 surveys of NNRTI PDR in adults conducted in 9 countries between 2000 and 2018, for a total of 14,567 individuals. All data used in the analysis is available to download from `https://github.com/jriou/nnrtipdr_sa`.

1.2 Data on four key indicators

For each of the nine countries, we obtained yearly estimates from UNAIDS regarding the number of adults living with HIV ($\mathbb{A}_t$), the number of adults living with HIV on ART ($\mathbb{B}_t$), and the number of AIDS-related deaths among adults ($\mathbb{C}_t$) for each year $t \in \{2000, \ldots, 2018\}$ [2]. This data constitute model-based estimates (using Spectrum or Thembisa [3, 4]) and not direct measurements. Data on the size of the adult population

(>15) of each country ($\mathbb{D}_t$) was obtained from the World Bank website [5]. Data for the year 1999 ($\mathbb{A}_0$ and $\mathbb{D}_0$) was also extracted to inform on initial conditions.

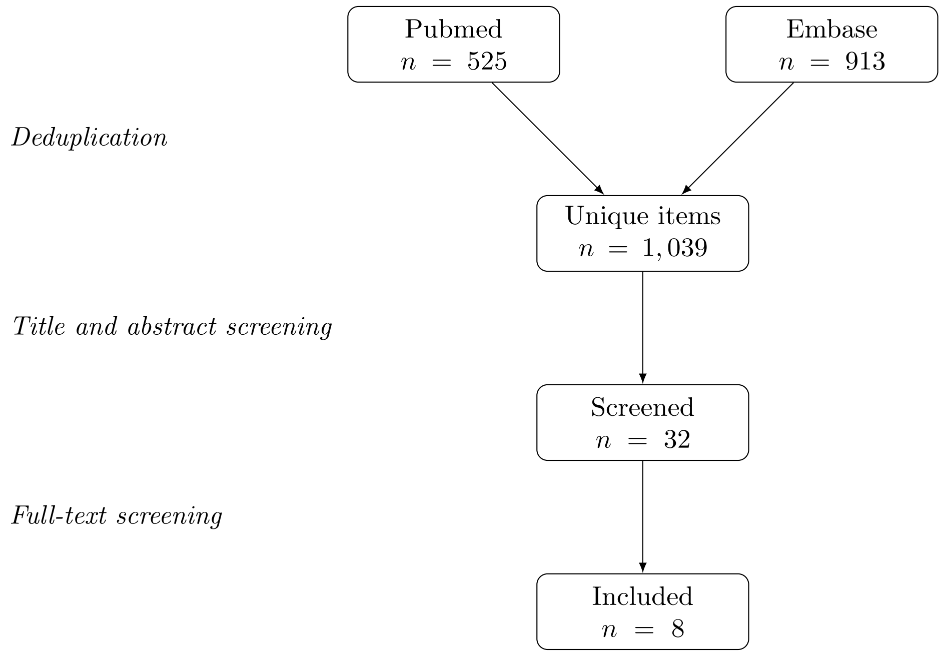

Figure 1: Flowchart of the update to the systematic review.

2 Additional model description

2.1 HIV transmission dynamics

We developed a deterministic, population-based HIV-1 transmission model using ordinary differential equations (ODEs) (figure 2). Our objective was to jointly model four key indicators of the local HIV epidemic (HIV prevalence, ART coverage, AIDS-related mortality, and population size) and the level of PDR for the adult population of a given country during the period 2000–2018.

The adult population of each country is divided into six compartments: susceptible to HIV-1 (S), infected with a NNRTI-sensitive strain of HIV-1 and untreated (I), infected with a NNRTI-sensitive strain and treated with first-line ART (T), infected with a NNRTI-resistant strain and untreated (J), infected with a NNRTI-resistant strain and treated with first-line ART (virological failure, U), and infected with a NNRTI-resistant strain and treated with second-line ART (V). The model considers the following dynamics:

Transitions between compartments reflect the following mechanisms:

- **Transmission**. Susceptible individuals may acquire a NNRTI-sensitive strain of HIV-1 from I, or a NNRTI-resistant strain from J or U, according to a transmission rate β. This assumes that (1) individuals infected with a NNRTI-sensitive strain and treated with first-line ART (compartment T) or individuals infected with a NNRTI-resistant strain and treated with second-line ART (compartment V) do not transmit the virus at a meaningful level[6] and (2) there is no fitness cost associated with the transmission of NNRTI-resistant strains [7].

- **First-line, NNRTI-based treatment**. The progressive roll-out of NNRTI-based ART from the early 2000s is modeled using a logistic growth function. This feature is implemented as follows: a maximum treatment rate τ is constrained by an indicator variable $f(t,\nu,\xi)$:

$$f(t,\nu,\xi) = \frac{1}{1+e^{-\xi(t-\nu)}} \tag{1}$$

which takes as input ν the time until treatment reaches 50% of its maximal value, and ξ the steepness of the logistic curve. This rate applies to both I and J compartments, assuming that individuals infected with a NNRTI-sensitive or NNRTI-resistant strain of HIV-1 are equally likely to initiate ART as drug resistance tests are not performed in routine.

- **Emergence of *de novo* NNRTI resistance**. Individuals infected by a NNRTI-sensitive strain and under first-line ART (compartment T) may acquire resistance to NNRTI with a rate ω in response to the selective pressure created by the treatment. As the model does not differentiate between virological suppression, treatment failure or non-adherence (because no data on these aspects could be found for each country over the period), ω is interpreted as a compound measure of all phenomenons leading to the occurrence of drug resistance mutations under treatment including poor ART adherence, low patient retention, quality of ART service delivery and unreliable supply chains.
- **Second-line, NNRTI-free ART**. Adults who fail first-line ART due to NNRTI resistance (compartment U) may be switched to second-line ART regimens that do not include with a rate κ.
- **Demography**. New susceptible adults enter the population at rate η. All adults can die from background mortality at rate μ, which we fix to the inverse of the life expectancy in adults in each country. HIV-1 infected adults without treatment (compartments I and J) or who fail firt-line ART without being switched to second line (compartment U) may die from AIDS-related mortality at rate δ.

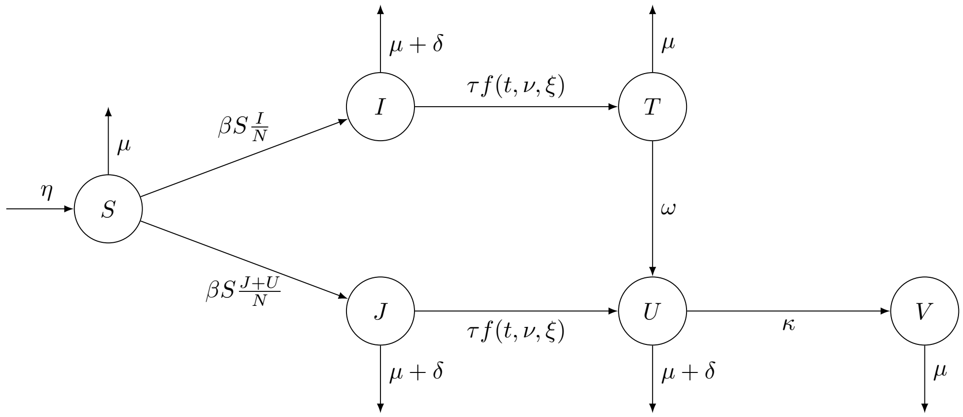

Figure 2: Schematic illustration of the HIV transmission model.

We select $t_0 = 1999$ as the starting date. Initial conditions are set-up using data for the year 1999 (population size $\mathbb{D}_0$ and HIV prevalence $\mathbb{A}_0$). An additional parameter ι is introduced to represent the initial proportion of individuals infected with a resistant strain:

$$
\begin{aligned}
S(t_0) &= \mathbb{D}_0 - \mathbb{A}_0 \\
I(t_0) &= (1 - \iota)\ \mathbb{A}_0 \\
J(t_0) &= \iota\ \mathbb{A}_0 \\
T(t_0) &= 0 \\
U(t_0) &= 0 \\
V(t_0) &= 0
\end{aligned} \tag{2}
$$

The ODE system thus relies upon seven parameters related to the natural history of HIV in a country $\theta_1 = \{\beta, \tau, \nu, \xi, \kappa, \eta, \delta\}$ (μ being fixed to a chosen value) and two parameters related to resistance to NNRTI $\theta_2 = \{\omega, \iota\}$. Upon solving the ODE system with numerical methods [8], we obtain as output the number of individuals in each compartment $S(t)$, $I(t)$, $J(t)$, $T(t)$, $U(t)$ and $V(t)$ for each year $t \in \{2000, \cdots, 2018\}$.

In addition to these, we introduce three dummy compartments that do not have any influence on the system dynamics but only record the cumulative incidence of AIDS-related deaths C_D:

$$\frac{dC_D}{dt} = \delta(I + J + U) \tag{3}$$

and the cumulative incidence of new treatment initiations of individuals infected a with NNRTI-sensitive (C_T) or a resistant strain (C_U):

$$\begin{aligned}\frac{dC_T}{dt} &= \tau f(t,\nu,\xi)I \\ \frac{dC_U}{dt} &= \tau f(t,\nu,\xi)J\end{aligned} \tag{4}$$

Outputs from the ODE system are further transformed into a set of values $\{a_t,\ldots,e_t\}$ matching the structure of the data, as shown in Table 3.

Table 3: Data and correspondence with model outputs.

Data	Interpretation	Corresponding ODE output
$\mathbb{A}_t$	Number of adults living with HIV at year t	$a_t = I(t) + T(t) + J(t) + U(t) + V(t)$
$\mathbb{B}_t$	Number adults on first-line or second-line ART at year t	$b_t = T(t) + U(t) + V(t)$
$\mathbb{C}_t$	Number of AIDS-related deaths among adults at year t	$c_t = C_D(t) - C_D(t-1)$
$\mathbb{D}_t$	Adult population size at year t	$d_t = S(t) + I(t) + T(t) + J(t) + U(t) + V(t)$
$\mathbb{E}_t/\mathbb{N}_t$	Proportion of ART naïve adults infected with an NNRTI-resistant strain in a survey of size conducted at year t	$e_t = \frac{C_U(t)-C_U(t-1)}{C_U(t)-C_U(t-1)+C_T(t)-C_T(t-1)}$

2.2 Hierarchical structure

The previous section described the model in one country. In order to extend the model to 9 countries, we consider 9 parallel and hierarchically-structured ODE systems. In country j, parameters related to the natural history of HIV $\theta_{1,j}$ are considered as independent across countries. We impose a hierarchical structure by country on the parameters related to NNRTI resistance $\theta_{2,j}$, so that:

$$\begin{aligned}\omega_j &\sim \text{lognormal}(\mu_\omega,\sigma_\omega) \\ \log\frac{\iota_j}{1-\iota_j} &\sim \mathcal{N}(\mu_\iota,\sigma_\iota)\end{aligned} \tag{5}$$

where $\theta_3 = \{\mu_\omega,\sigma_\omega,\mu_\iota,\sigma_\iota\}$ are hyperparameters governing the between-country distribution of the "random-effects" ω_j and ι_j. We can consider the full ODE model as a function with as inputs the parameters and as outputs the model-predicted HIV-1 prevalence, ART coverage, AIDS-related mortality, population size and NNRTI PDR in each country for the period 2000–2018:

$$g(\theta_{1,j},\theta_{2,j},\theta_3) = \{a_{j,t},b_{j,t},c_{j,t},d_{j,t},e_{j,t}\} \tag{6}$$

2.3 Statistical inference

The outputs of the ODE system $\{a_{j,t},\ldots,e_{j,t}\}$ at time t in country j are fitted to data in order to estimate the parameters .

For the four key indicators of the HIV-1 epidemic, we use a multivariate normal likelihood after a variance-stabilizing square-root transformation [9], which can be expressed as:

$$\Pr(\mathbb{A}_{j,t},\mathbb{B}_{j,t},\mathbb{C}_{j,t},,\mathbb{D}_{j,t} \mid \theta_{1,j},\theta_{2,j},\theta_3,\Sigma_j) = \mathcal{N}\left(\sqrt{\begin{bmatrix}\mathbb{A}_{j,t}\\ \mathbb{B}_{j,t}\\ \mathbb{C}_{j,t}\\ \mathbb{D}_{j,t}\end{bmatrix}} \;\middle|\; \sqrt{\begin{bmatrix}a_{j,t}\\ b_{j,t}\\ c_{j,t}\\ d_{j,t}\end{bmatrix}},\ \Sigma_j\right) \tag{7}$$

Here, Σ_j is a diagonal covariance matrix:

$$\Sigma_j = \begin{bmatrix} \sigma^2_{j,a} & 0 & 0 & 0 \\ 0 & \sigma^2_{j,b} & 0 & 0 \\ 0 & 0 & \sigma^2_{j,c} & 0 \\ 0 & 0 & 0 & \sigma^2_{j,d} \end{bmatrix} \tag{8}$$

and $\{\sigma^2_{j,a}, \cdots, \sigma^2_{j,d}\}$ are variance parameters, with covariance parameters being fixed to 0.

Survey data on NNRTI PDR has a different structure. Considering survey i, data consists of the number of ART-naïve adults with NNRTI resistance $\mathbb{E}_i$, the sample size $\mathbb{N}_i$, the year of sampling $\mathbb{T}_i$ and the country of sampling $\mathbb{J}_i$. We use a binomial likelihood of the following form:

$$\Pr(\mathbb{E}_i, \mathbb{N}_i, \mathbb{T}_i, \mathbb{J}_i | \theta_{1,\mathbb{J}_i}, \theta_{2,\mathbb{J}_i}, \theta_3, \Sigma_{\mathbb{J}_i}) = \text{Binom}(\mathbb{E}_i \mid \mathbb{N}_i, e_{\mathbb{J}_i,\mathbb{T}_i}) \tag{9}$$

The full likelihood can be expressed as:

$$\begin{aligned} \Pr(\mathbb{A}, \mathbb{B}, \mathbb{C}, \mathbb{D}, \mathbb{E}, \mathbb{N}, \mathbb{T}, \mathbb{J} | \theta_1, \theta_2, \theta_3, \Sigma) = &\prod_j \prod_t \Pr(\mathbb{A}_{j,t}, \mathbb{B}_{j,t}, \mathbb{C}_{j,t}, , \mathbb{D}_{j,t} \mid \theta_{1,j}, \theta_{2,j}, \theta_3, \Sigma_j) \times \\ &\prod_i \Pr(\mathbb{E}_i, \mathbb{N}_i, \mathbb{T}_i, \mathbb{J}_i | \theta_{1,\mathbb{J}_i}, \theta_{2,\mathbb{J}_i}, \theta_3, \Sigma_{\mathbb{J}_i}) \end{aligned} \tag{10}$$

Table 4: Parameters of the model and chosen prior distributions.

Parameter	Interpretation	Prior	Unit
Country-level parameters			
β_j	Transmission rate	Expon(5)	y^{-1}
τ_j	Maximum treatment rate	Expon(5)	y^{-1}
ν_j	Delay until the effective treatment rate reaches 50% of its maximum	Expon(0.5)	y
ξ_j	Slope of logistic function	Beta(2, 2)	-
κ_j	Rate of switching to second-line ART	Expon(5)	y^{-1}
δ_j	AIDS-related mortality rate	Expon(5)	y^{-1}
η_j	Adult population growth rate	Expon(5)	y^{-1}
ω_j	Rate of treatment failure due to NNRTI resistance	Gamma($\mu_\omega, \sigma_\omega$)	y^{-1}
ι_j	Initial proportion of NNRTI resistance in 1999	$\mathcal{N}(\text{logit}(\mu_\iota), \sigma_\iota)$	y^{-1}
Hyperparameters			
μ_ω	Location of the distribution of ω_j across countries	Expon(5)	y^{-1}
σ_ω	Scale of the distribution of ω_j across countries	Expon(20)	y^{-1}
μ_ι	Location of the distribution of ι_j across countries	Beta(1, 9)	y^{-1}
σ_ι	Scale of the distribution of ι_j across countries	Expon(5)	y^{-1}
Variance parameters			
Σ_j	Covariance matrix, reparametrized into $\{\sigma_a, \cdots, \sigma_d\}$	-	-
$\{\sigma_{j,a}, \cdots, \sigma_{j,d}\}$	Scales for the multivariate normal distribution	Expon(1)	-

2.4 Prior predictive checks

We selected weakly-informative priors distributions for all parameters (table 4). We conducted prior predictive checks [?] to ensure that the chosen priors limited the range of explored parameter space to sensible values. For this, we generated fake data from the prior distributions and checked that the resulting values were compatible with actual data, without constraining parameter inference to specific values. The results are shown in figures 3 4. The large range of values generated from the prior distributions corresponds to the possibilities implied by our choice of priors. This is an indication that our assumptions regarding the priors did not influence parameter inference, and that they can be qualified as "weakly-informative".

2.5 Implementation and code

The model was implemented in Stan 2.18.2 [10]. The joint posterior distribution of the parameters was explored with the No-U-Turn Sampler (NUTS), a variant of Hamiltonian Monte Carlo [**?**]. The model code, as well as posterior samples and R scripts are available on `https://github.com/jriou/nnrtipdr_sa`.

References

[1] Ravindra K Gupta, John Gregson, Neil Parkin, Hiwot Haile-Selassie, Amilcar Tanuri, Liliana Andrade Forero, Pontiano Kaleebu, Christine Watera, Avelin Aghokeng, Nicholus Mutenda, et al. Hiv-1 drug resistance before initiation or re-initiation of first-line antiretroviral therapy in low-income and middle-income countries: a systematic review and meta-regression analysis. *The Lancet infectious diseases*, 18(3):346–355, 2018.

[2] UNAIDS. Global HIV and AIDS statistics. `http://www.unaids.org/en/resources/fact-sheet`. Accessed: 2019-04-29.

[3] John Stover, Tim Brown, and Milly Marston. Updates to the spectrum/estimation and projection package (epp) model to estimate hiv trends for adults and children. *Sex Transm Infect*, 88(Suppl 2):i11–i16, 2012.

[4] Leigh Johnson, Rob Dorrington, Thomas Rehle, Sean Jooste, Linda-Gail Bekker, Melissa Wallace, Landon Myer, and Andrew Boulle. Thembisa version 1.0: A model for evaluating the impact of hiv/aids in south africa. *Centre for Infectious Disease Epidemiology and Research Working Paper February*, 2014, 2014.

[5] World Bank. Health Nutrition and Population Statistics. `https://data.worldbank.org/`. Accessed: 2019-04-29.

[6] Virginie Supervie, Jean-Paul Viard, Dominique Costagliola, and Romulus Breban. Heterosexual risk of hiv transmission per sexual act under combined antiretroviral therapy: systematic review and bayesian modeling. *Clinical Infectious Diseases*, 59(1):115–122, 2014.

[7] Susan J Little, Simon DW Frost, Joseph K Wong, Davey M Smith, Sergei L Kosakovsky Pond, Caroline C Ignacio, Neil T Parkin, Christos J Petropoulos, and Douglas D Richman. Persistence of transmitted drug resistance among subjects with primary human immunodeficiency virus infection. *Journal of virology*, 82(11):5510–5518, 2008.

[8] John R Dormand and Peter J Prince. A family of embedded runge-kutta formulae. *Journal of computational and applied mathematics*, 6(1):19–26, 1980.

[9] Guan Yu. Variance stabilizing transformations of poisson, binomial and negative binomial distributions. *Statistics & Probability Letters*, 79(14):1621–1629, 2009.

[10] Bob Carpenter, Andrew Gelman, Matthew D Hoffman, Daniel Lee, Ben Goodrich, Michael Betancourt, Marcus Brubaker, Jiqiang Guo, Peter Li, and Allen Riddell. Stan: A probabilistic programming language. *Journal of statistical software*, 76(1), 2017.

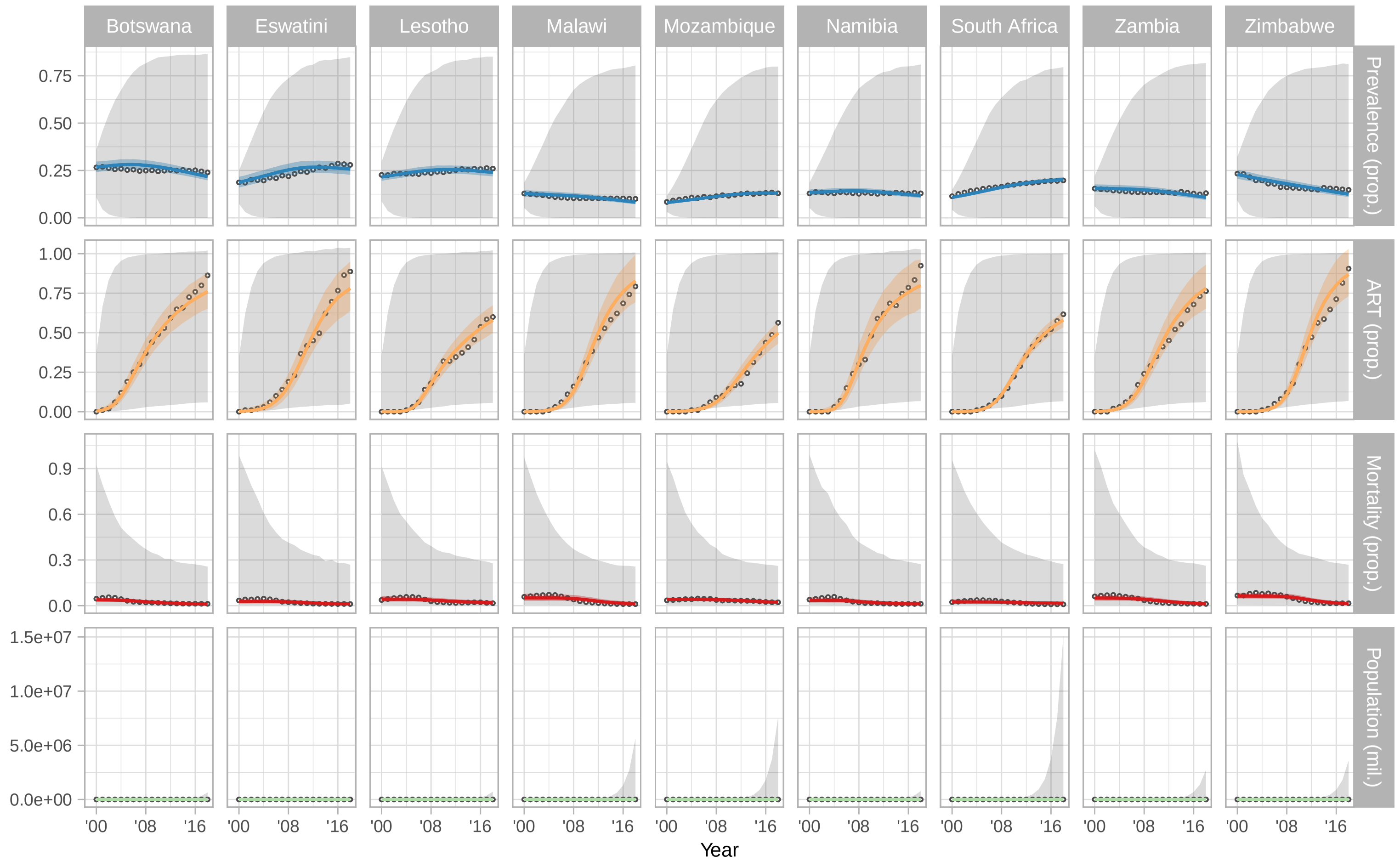

Figure 3: Prior predictive check for indicator data. Grey areas correspond to the 95% central range of values generated from the prior distributions. Colored areas correspond to the model fit. Circles are data points.

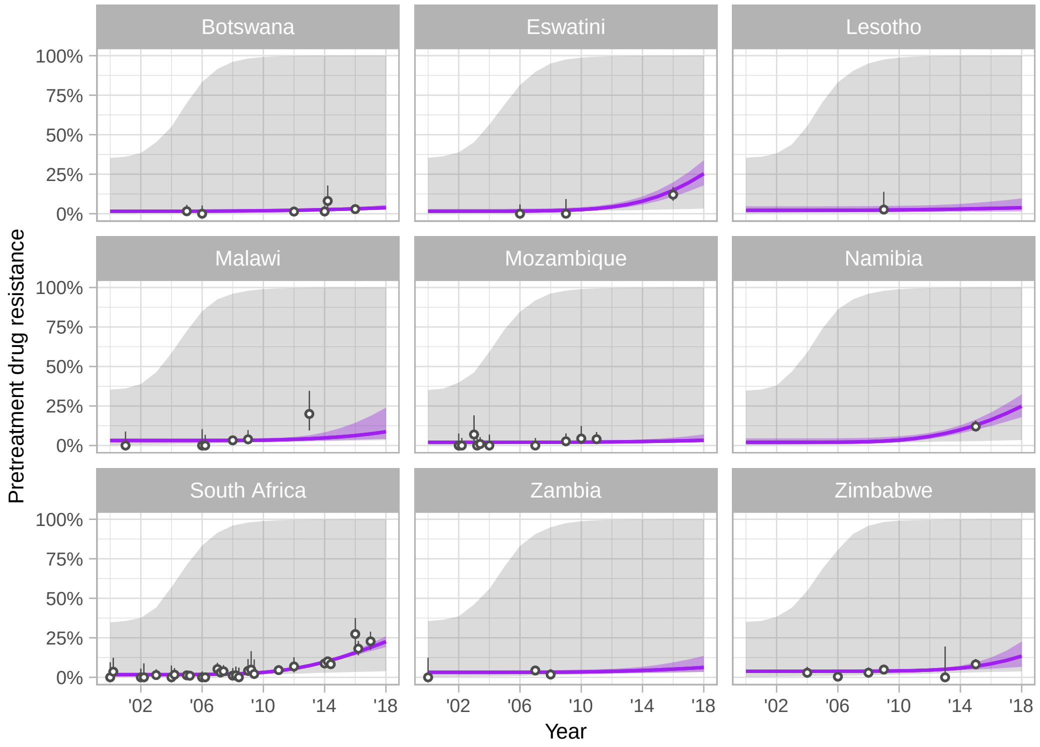

Figure 4: Prior predictive check for NNRTI PDR data. Grey areas correspond to the 95% central range of values generated from the prior distributions. Colored areas corresponds to the model fit. Circles are data points.
